# Grazing offsets the stimulating effects of nitrogen addition on soil CH_4_ emissions in a meadow steppe in Northeast China

**DOI:** 10.1371/journal.pone.0225862

**Published:** 2019-12-02

**Authors:** Rongrong Ren, Wanling Xu, Mingming Zhao, Wei Sun

**Affiliations:** Key Laboratory for Vegetation Ecology, Ministry of Education Institute of Grassland Science, Northeast Normal University, Changchun, Jilin Province, China; Chinese Academy of Sciences, CHINA

## Abstract

Grazing is the most common land use type for grasslands, and grazing may alter the impacts of the predicted enhancement of nitrogen deposition on soil CH_4_ flux. To understand the effects of nitrogen addition, grazing, and their interactions on soil CH_4_ flux, we conducted a field study on CH_4_ flux in a meadow steppe in Northeast China from 2017 to 2018. We measured the soil CH_4_ flux and soil physiochemical and vegetation parameters. The studied meadow steppe soil acted as a CH_4_ source due to the legacy effects of an extreme rainfall event. During the experimental period, the average CH_4_ fluxes were 7.8 ± 1.0, 5.8 ± 0.5, 9.3 ± 0.9 and 7.6 ± 0.6 μg m^-2^ h^-1^ for the CK (control), G (grazing), N (nitrogen addition) and NG (grazing and nitrogen addition) treatments, respectively. The cumulative CH_4_ fluxes were 24.9 ± 2.6, 11.5 ± 4.9, 28.8 ± 4.2 and 17.8 ± 3.5 μg m^-2^ yr^-1^ for the CK, G, N and NG treatments, respectively. The N addition increased the average CH_4_ flux by 19%, and the grazing treatment reduced it by 25%. The soil CH_4_ flux was positively correlated with the 0–10 cm soil water filled pore space (*P* < 0.01), soil NH_4_^+^-N (*P* < 0.01) and soil NO_3_^-^-N (*P* < 0.01), but negatively correlated with the 0–10 cm soil temperature (*P* < 0.01), except for the sampling dates that were strongly influenced by the extreme rainfall event. The average CH_4_ flux was significantly (*P* < 0.05) affected by the grazing and N addition treatments with the N addition treatment significantly (*P* < 0.05) increased the CH_4_ flux, whereas grazing significantly (*P* < 0.05) decreased the CH_4_ flux. Grazing offset the stimulating effects of N addition on CH_4_ flux, and there was no difference (*P* = 0.79) in the CH_4_ flux between the CK and NG plots. In summary, moderate grazing has the potential to reduce the negative impacts of N addition on CH_4_ flux and can increase the capacity of the soil CH_4_ sink in the studied meadow steppe.

## Introduction

As the second most important greenhouse gas, methane (CH_4_) has a global warming potential 34 times that of carbon dioxide (CO_2_) for the time horizon of 100 years and contributes approximately 25% to global warming [1]. The atmospheric CH_4_ concentration has increased from 722 ppb to 1803 ppb since the industrial revolution [1]. Soil CH_4_ flux is determined by the balance between methanogenic bacteria-associated CH_4_ production and methanotrophic bacteria-related CH_4_ consumption [2]. Other than soil microbial activity, CH_4_ flux is also influenced by soil physiochemical conditions, such as moisture, temperature, pH, organic C content and inorganic N content [3,4]. In an African tropical montane region, the combined contribution of soil water content and bulk density explained over 49% of the total variation in soil CH_4_ fluxes [5]. These abiotic and biotic factors are likely to be affected by global change factors (e.g., nitrogen addition, altered precipitation regime) as well as land use patterns and extent (e.g., grazing), and subsequently modify soil CH_4_ flux [6,7]. Indeed, there is evidence that CH_4_ uptake differs significantly among land-cover types, suggesting that CH_4_ uptake may respond differently in different land-covers or land-use change scenarios [8].

Nitrogen (N) deposition has continued to increase since the last century, mainly due to fossil fuel combustion [9]. The impacts of enhanced N input on CH_4_ flux have attracted much attention, but the results are not consistent [10–12]. The response of soil CH_4_ flux to N addition is still uncertain and is mainly determined by the forms and rates of N addition and soil properties [13]. The results of a meta-analysis showed that N addition reduced CH_4_ uptake by 38% [13]. It has often been reported that CH_4_ uptake is significantly reduced by long-term N addition, which can change soil pH and nitrate N content [14]. There is evidence that long-term N addition may increase the availability of soil NH_4_^+^ and soil NO_3_^-^, thus changing the activity of methanogenic bacteria and methanotrophic bacteria, and consequently affecting soil CH_4_ flux [10]. However, some studies have suggested that the content of soil NH_4_^+^, but not NO_3_^-^, is the dominant factor controlling CH_4_ oxidation rates [15]. High soil NH_4_^+^ concentrations may reduce the oxidation capacity of soils for atmospheric CH_4_, thus decreasing the net flux of CH_4_ from the atmosphere to the soil [15]. N addition can also promote plant growth and trigger changes in soil properties, such as soil moisture and soil temperature, which may also affect CH_4_ flux. In addition to the uncertainty concerning the effects of N addition on CH_4_ flux, land use patterns, such as grazing, may also interact with N addition and alter CH_4_ flux.

Grazing is the major land use type for grasslands, and its impact on the production and consumption of CH_4_ flux has been reported widely [16–18]. Grazing alters CH_4_ flux by affecting plant productivity, soil properties and soil microbial activity [19–21]. First, livestock trampling changes the soil bulk density and weakens the diffusivity of CH_4_ and O_2_ across the soil profile [22]. Second, grazing can reduce the aboveground biomass [23] and litter biomass, which can affect soil temperature and soil moisture. Third, grazing may alter the composition and activity of methanogenic bacteria and methanotrophic bacteria [24]. The impacts of grazing on CH_4_ highly depend on grazing intensity, grazing time and grazing site [18]. Grazing may offset N deposition-associated negative effects on CH_4_ flux by changing soil N availability and soil physical characteristics. However, little information is available regarding the interactive effects of N addition and grazing on soil CH_4_ flux.

Simulated atmospheric N deposition and/or grazing experiments have been conducted in many natural ecosystems. However, the impact of N deposition and grazing on the production or consumption of CH_4_ are not well understood. To understand the effects of N addition, grazing, and their interactions on grassland CH_4_ flux, we conducted a field study on CH_4_ flux in a meadow steppe in Northeast China. The main aims of this study were (1) to investigate the seasonal dynamics of CH_4_ flux and unravel the underlying mechanisms in the meadow steppe; (2) to examine the effects of N addition, grazing and their interactions on soil CH_4_ flux. We hypothesized that (1) the soil CH_4_ flux in the studied meadow steppe would display strong seasonal variation and may be highly influenced by the WFPS (water filled pore space), topsoil temperature and inorganic N content; (2) the N addition treatment would increase the CH_4_ flux, whereas grazing would decrease the CH_4_ flux and the grazing treatment would offsets the stimulating effects of N addition on the CH_4_ flux.

## Materials and methods

### Ethics statement

No specific permissions were required to conduct research at the field site, because the Songnen Grassland Ecological Research Station is a department of the Northeast Normal University. No specific permissions were required for the study either, as it was conducted in accordance with the guidelines set by the Northeast Normal University. No specific permissions were required for the locations or the activities. No location was privately owned or protected in any way, and the field studies did not involve endangered or protected species. The cattle used on the experiment were rented from a ranch. During the experimental grazing period, the cattle had access to food and water. After the experimental grazing, the cattle grazed freely in the surrounding grasslands.

### Site description

This experiment was conducted in the Songnen meadow steppe, which is located in western Jilin Province, Northeast China (44°40′-44°44′ N, 123°44′-123°47′ E). The study area is influenced by a temperate semiarid monsoon climate. The annual average temperature is 6.4°C (1950–2004), and the frost-free period is 150 days. The average annual precipitation is 471 mm (1950–2004), with over 70% occurring from June to August [25]. The vegetation is dominated by *Leymus chinensis*, a C_3_ perennial rhizomatous grass. *Phragmites australis* (a perennial C_3_ plant), *Chloris virgata* (an annual C_4_ plant) and *Kalimeris integrifolia* (a perennial C_3_ plant) are also abundant [26]. The vegetation coverage ranged from 50% to 90%, with 100–360 g m^-2^ of aboveground biomass in the peak biomass season [27]. The soil in the study area is saline-alkaline, which is equivalent to an Aqui-Alkalic Halosol based on the Chinese soil classification or a Salic Solonetz in the World Reference Base for Soil Resources (WRB) [28]. The studied soil had an organic carbon content of 2.0% and a total nitrogen content of 0.15%, and the soil pH ranged from 8.0 to 9.0 [29]. The available soil phosphorus content was 2.5 mg kg^- 1^. The soils contained high contents of free sodium bicarbonate (NaHCO_3_) and sodium carbonate (Na_2_CO_3_) [27].

### Experimental design

In 2010, we fenced a grassland with an area of 400 m × 100 m in the experimental site. Grazing by large herbivores and mowing were excluded from the fenced area. In August 2015, 4 blocks (100 m × 100 m each) with similar vegetation compositions were established within the fenced grassland. We laid out 4 plots (30 m × 30 m each) in each block. There were no significant differences in the vegetation characteristics among the blocks and plots. Within each block, we randomly assigned one of four treatments (control, CK; grazing, G; nitrogen addition, N; and grazing and nitrogen addition, NG) to each plot. The grazing and nitrogen addition treatments were initiated in May 2016. For each month of the growing season (May to September), each of the G and NG plots received one-day (06:00 AM to 10:00 AM) moderate grazing (approximately 50% of the aboveground biomass was consumed by herbivores) by adult Simmental cattle [30]. To minimize stocking rate differences between the G and NG treatments, 14 cattle and 18 cattle were allowed to graze in the G and NG plots, respectively. This assignment greatly reduced stocking rate differences between the G and NG treatments. One week after the grazing treatment, urea (2 g m^-2^) was manually spread on the N and NG plots. For each year of the experiment, urea was applied five times (once per month from May to September), which resulted in an N addition rate of 10 g m^-2^ yr^-1^.

### Sampling and measurement of CH_4_ flux

Soil CH_4_ fluxes were measured from the beginning of April 2017 through the end of September 2018. Gas sampling was conducted once per week during the growing season (May-September), twice a month during the non-growing season (April, October, and November), once a month during the winter (December, January, and February), and every three days during the freezing and thawing period (March).

The static opaque chamber method was used for the measurements of soil CH_4_ flux [24]. The static chamber consisted of two parts: a stainless steel (length × width × height = 30 cm × 30 cm × 15 cm) base and a box (length × width × height = 30 cm × 30 cm × 60 cm) made of polypropylene. To prevent the influence of direct radiative heating during the sampling period, the outside of the chamber was covered by reflective aluminium foil. A fan was installed inside the chamber to mix the air. The stainless steel base had a groove to connect with the chamber, and during the gas sampling period, the chamber was sealed by the addition of water to the groove. The steel base was inserted into the soil surface (10 cm) before the gas sampling.

During each gas sampling campaign, gas samples were collected between 09:00 AM and 11:00 AM (China Standard Time, CST) [31]. The chambers were closed for an hour, and gas samples (200 ml) were collected every 20 min using plastic syringes. The temperature inside the chamber was recorded at the same time using an electronic thermometer (DT-1, Jingchuang, China). The collected gas samples were stored in previously evacuated gas sampling bags (200 ml) before the laboratory CH_4_ concentration measurements. In each plot, we randomly selected six points for gas sampling and sampled these points during each of the subsequent gas sampling campaigns. The CH_4_ concentration was determined using a CH_4_/N_2_O gas analyser (Model 913–1054, Los Gatos Research, USA) within one week of field sampling.

CH_4_ flux was calculated as follows:

$$F=\rho \times \frac{V}{A}\times \frac{\Delta c}{\Delta t}\times \frac{273}{273+T}$$

where *F* is the CH_4_ flux (mg m^-2^ h^-1^); *ρ* is the density of CH_4_ (mg m^-3^) under standard conditions; *V* (m^3^) and *A* (m^2^) are the volume and base area of the opaque chamber, respectively; *Δc/Δt* is the rate of change in the CH_4_ concentration per hour; and *T* (°C) is the average temperature inside the chamber [32]. Negative flux values indicate CH_4_ uptake from the atmosphere, and positive flux values indicate CH_4_ emission to the atmosphere [33].

### Soil temperature and soil water filled pore space

Soil temperature and soil water-filled pore space (WFPS) at a depth of 0–10 cm were monitored during each gas sampling event near the gas sampling points. Soil temperature was measured by a soil temperature probe (TPG-21, Tuopu, China) with six replicates in each plot.

For the measurement of WFPS, we used the ring knife method to collect the soil samples (50 cm^-3^). The collected soil samples were placed in aluminium boxes, and the fresh weight was measured. The dry weight was measured after oven-drying at 105°C to a constant weight [34]. The volumetric soil moisture was calculated as the water loss divided by the soil core volume (50 cm^-3^). The soil bulk density was calculated by the ratio between the dry weight and the soil core volume. Based on the volumetric soil moisture, soil bulk density, and a particle size density of 2.65 g cm^-3^ [35], the WFPS was calculated as follows:

WFPS=volumetricsoilmoisture/(1−bulkdensity/2.65)

### Precipitation

Precipitation was measured using an RG2-M sensor (Onset Computer Corporation, Bourne, MA, USA) 0.2 km distant from the experimental site.

### Soil sampling

Using a soil auger (2.5 cm in diameter), soil samples (0–10 cm) were collected near the gas sampling point once per month from April 2017 to September 2018. For each gas sampling point, we sampled soils from 6 locations and thoroughly mixed them together into a combined sample during each soil sampling campaign. The mixed soils were then sieved (2 mm) to remove roots and stones in the field and kept in a refrigerator at -20°C before analysis.

### Soil NH_4_^+^-N, soil NO_3_^-^-N and soil pH

Ten grams of moist soil were placed in an Erlenmeyer flask and extracted with 50 ml KCl (2 M) and shaken for 1 h. After shaking, the samples were allowed to settle and were filtered through filter paper; then, the samples were stored at -20°C before analysis. The soil NH_4_^+^-N and soil NO_3_^—^N contents were measured by a continuous flow autoanalyser (FUTURA, AMS Alliance, Italia). Soil pH was measured by mixing 10 g soil with water in a 1:5 ratio. The resulting slurry was stirred for 1 h and then allowed to sit for another hour before pH was measured using a pH probe (Phs-3c, Shanghai, China) [36].

### Vegetation survey and biomass measurement

A vegetation survey and biomass measurements were conducted once per year in 2017 and 2018. In each plot, the number of plant species and the number of individuals of each species were counted in six randomly placed quadrats (50 cm × 50 cm) at the end of July (approximately two weeks after the grazing treatment in July). The aboveground biomass (AGB) was harvested after the vegetation survey. Litter was also collected. The belowground biomass (BGB) was determined by washing roots out of a soil core with a diameter of 7.0 cm collected at a depth of 0–10 cm [37]. One soil core randomly collected in each quadrat was used for the vegetation survey. The AGB, BGB and litter samples were oven-dried at 65°C to a constant weight (approximately 48 h).

### Statistical analysis

The effects of G, N and their interactions on soil temperature, soil WFPS, soil inorganic N (NH_4_^+^-N and NO_3_^-^-N) content, average CH_4_ flux and cumulative CH_4_ flux were assessed with two-way ANOVAs. The influences of G, N, year and their interactions on the average CH_4_ flux, soil temperature, soil WFPS and biomass (AGB, BGB, and litter) were examined using three-way ANOVAs. Statistical significance was set at *P* < 0.05. Linear regression analyses were used to examine the dependence of CH_4_ flux on soil temperature, soil WFPS, soil NH_4_^+^-N, soil NO_3_^-^-N, soil pH and plant biomass. All statistical analyses were conducted using SPSS 22.0 software (SPSS, Inc., Chicago, USA), and the graphs were generated using SigmaPlot 12.5 software (Systat Software, Inc., Chicago, USA). The results were expressed as the mean value ± 1 standard error (n = 4).

## Results

### Environmental variables

**Fig 1:**
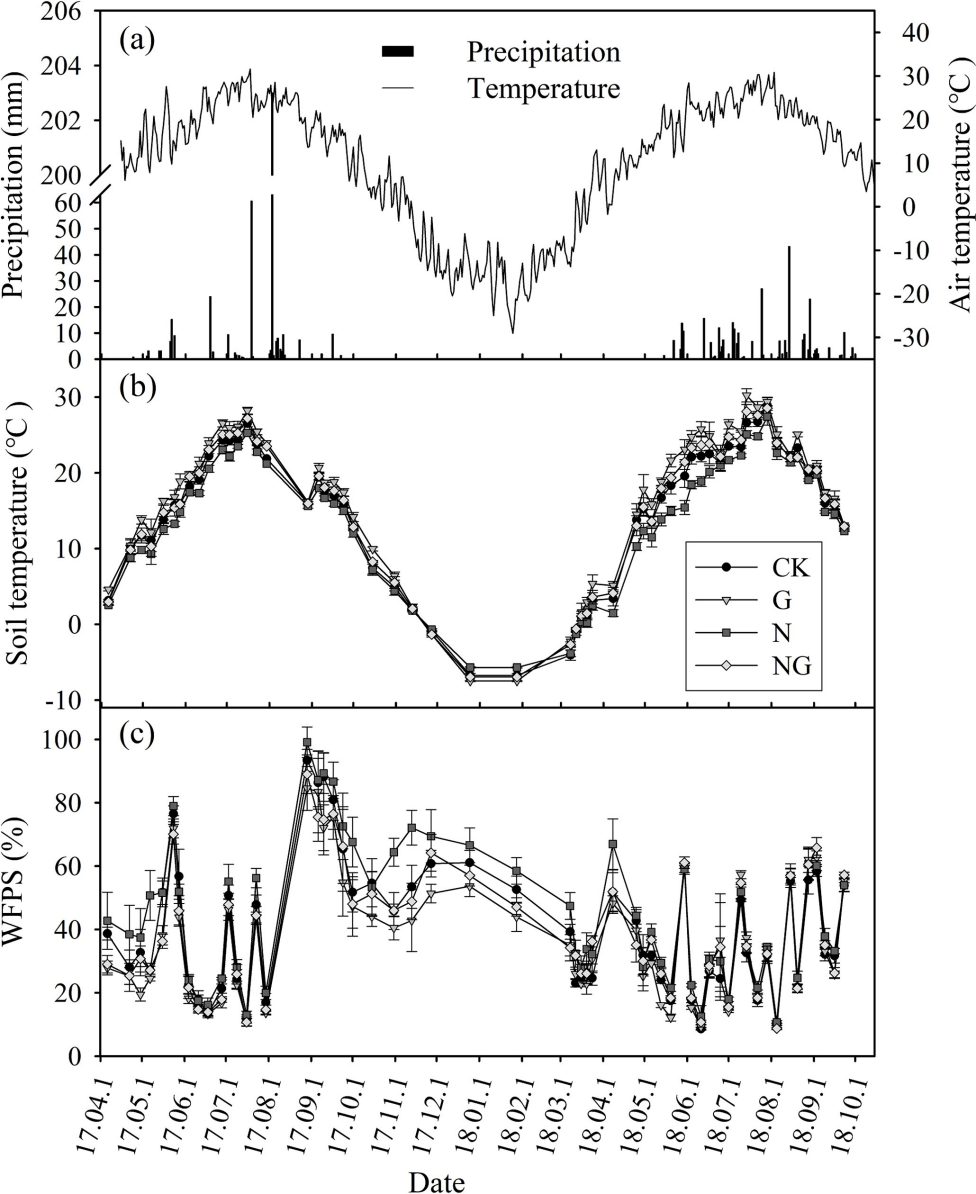
Variation in air temperature (a) and precipitation (a) from April 2017 to September 2018. Dynamics of soil temperature (b) and soil water filled pore space (c) in the different treatments (CK: control, G: grazing, N: nitrogen addition, NG: grazing and nitrogen addition) during the experimental period. Data are reported as the arithmetic mean ± 1 standard error (n = 4).

Over the experimental period, precipitation exhibited strong variation in timing and event size (Fig 1A). For example, there was an extreme rainfall event (203 mm) in August 2017 that accounted for 49% of the growing season precipitation. The amount of growing season precipitation (April—October) in 2018 (308 mm) was lower than that in 2017 (412 mm) (Fig 1A). The air temperature had clear seasonal patterns, being highest (31°C) in July and August and lowest (-29°C) in January (Fig 1A). The seasonality of the 0–10 cm soil temperature coincided with the seasonal patterns of air temperature. For both experimental years, the 0–10 cm soil temperature peaked (28°C) at the end of July (Fig 1B). During the growing season, the 0–10 cm soil WFPS varied substantially and was closely related to the precipitation events (Fig 1C).

### CH_4_ flux

**Fig 2:**
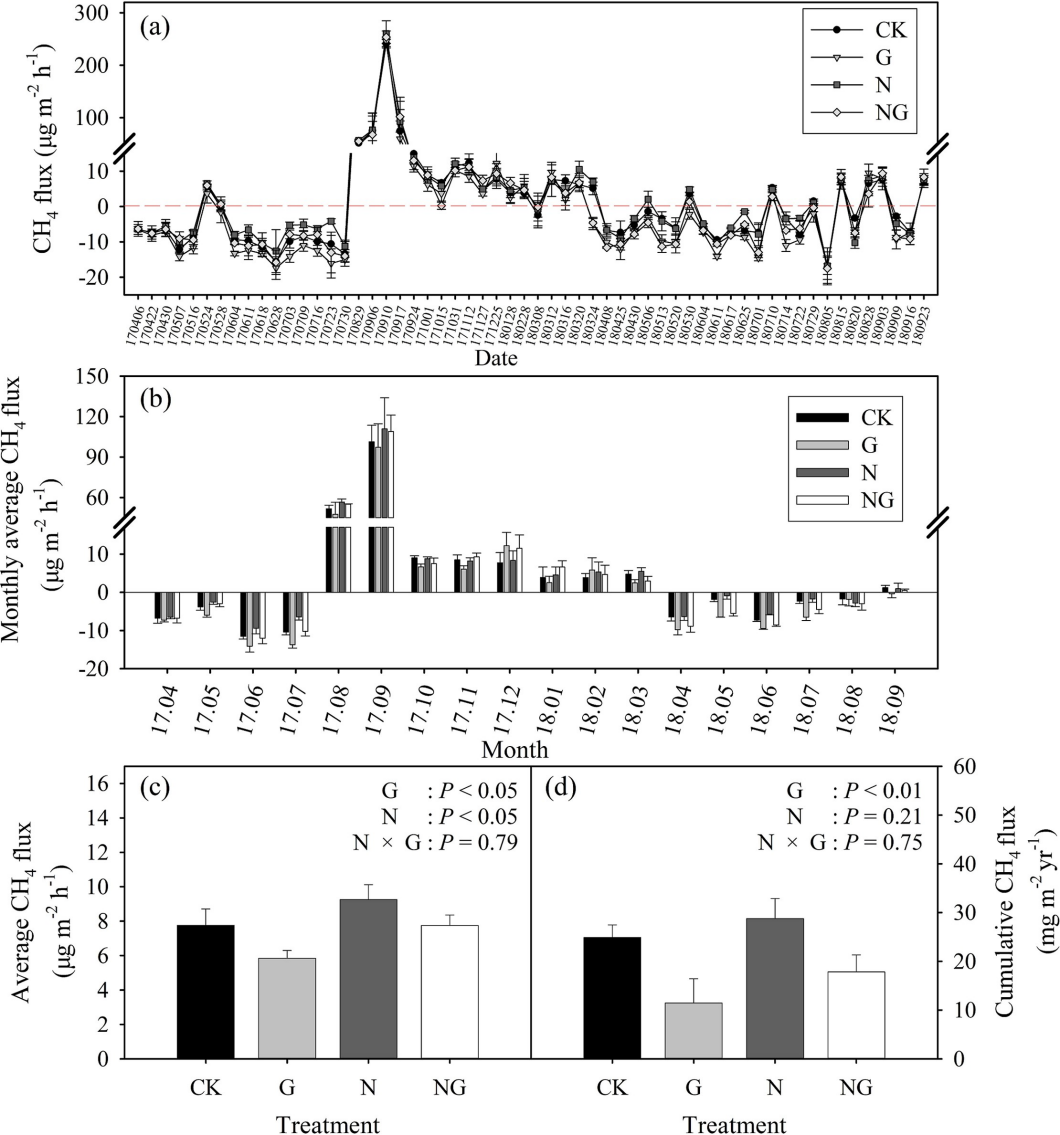
Seasonal variations (a), monthly average (b), annual average (c) and annual cumulative (d) of CH_4_ flux for the four experimental treatments (CK: control, G: grazing, N: nitrogen addition, NG: grazing and nitrogen addition) from April 2017 to September 2018). Results of two-way ANOVA for the effects of grazing, N addition and their interactions on average CH_4_ flux are provided. Data are reported as the arithmetic mean ± 1 standard error (n = 4).

The CH_4_ flux varied substantially during the experimental period, and there were no apparent differences in the variation patterns among the treatments (Fig 2A). From April to July 2017, CH_4_ fluxes were negative for most of the sampling dates, which indicated the studied grassland was a net CH_4_ sink during that time period. Following the extreme precipitation event (August 2017, 203 mm), there was a strong increase in CH_4_ emissions for all treatments, with the maximum CH_4_ emission rates (241.2 ± 8.1, 245.6 ± 4.3, 260.2 ± 24.6 and 253.6 ± 11.6 μg m^-2^ h^-1^ for the CK, G, N and NG treatments, respectively) were observed on 10 September. Then, the CH_4_ emission rate gradually decreased and tended to stabilize until March 2018. From April to September in 2018, the CH_4_ uptake rates in the studied grassland were low for most of the sampling dates; however, pulses of CH_4_ emissions were also observed occasionally (Fig 2A).

The monthly average CH_4_ fluxes were negative from April to July in 2017 and were positive from August 2017 to March 2018. The monthly average CH_4_ fluxes changed to negative after March 2018, and the studied grassland was a net CH_4_ sink from April to August 2018 (Fig 2B). During the experimental period, the average CH_4_ fluxes were 7.8 ± 1.0, 5.8 ± 0.5, 9.3 ± 0.9 and 7.6 ± 0.6 μg m^-2^ h^-1^ for the CK, G, N and NG treatments, respectively. The average CH_4_ flux was significantly (*P* < 0.05) affected by the grazing and N addition treatments. The nitrogen addition increased the average CH_4_ flux by 19%, and the grazing treatment reduced it by 25%. There were no significant interactive effects between the grazing and N addition treatments on average CH_4_ flux (*P* = 0.79) (Fig 2C).

The cumulative CH_4_ fluxes were 24.9 ± 2.6, 11.5 ± 4.9, 28.8 ± 4.2 and 17.8 ± 3.5 μg m^-2^ yr^-1^ for the CK, G, N and NG treatments, respectively. The cumulative CH_4_ flux was reduced significantly in the grazed plots (*P* < 0.01); however, it was not affected (*P* = 0.21) by the N addition. For all treatments, the cumulative CH_4_ fluxes (October 2017 to September 2018) were positive, indicating that CH_4_ emissions were higher than uptake (Fig 2D). The highest cumulative CH_4_ flux (October 2017 to September 2018) was observed in the N plots (28.8 ± 4.2 mg m^-2^ yr^-1^), whereas the lowest value was detected in the grazing plots (11.5 ± 4.9 mg yr^-1^).

### Aboveground biomass, belowground biomass and litter biomass

**Fig 3:**
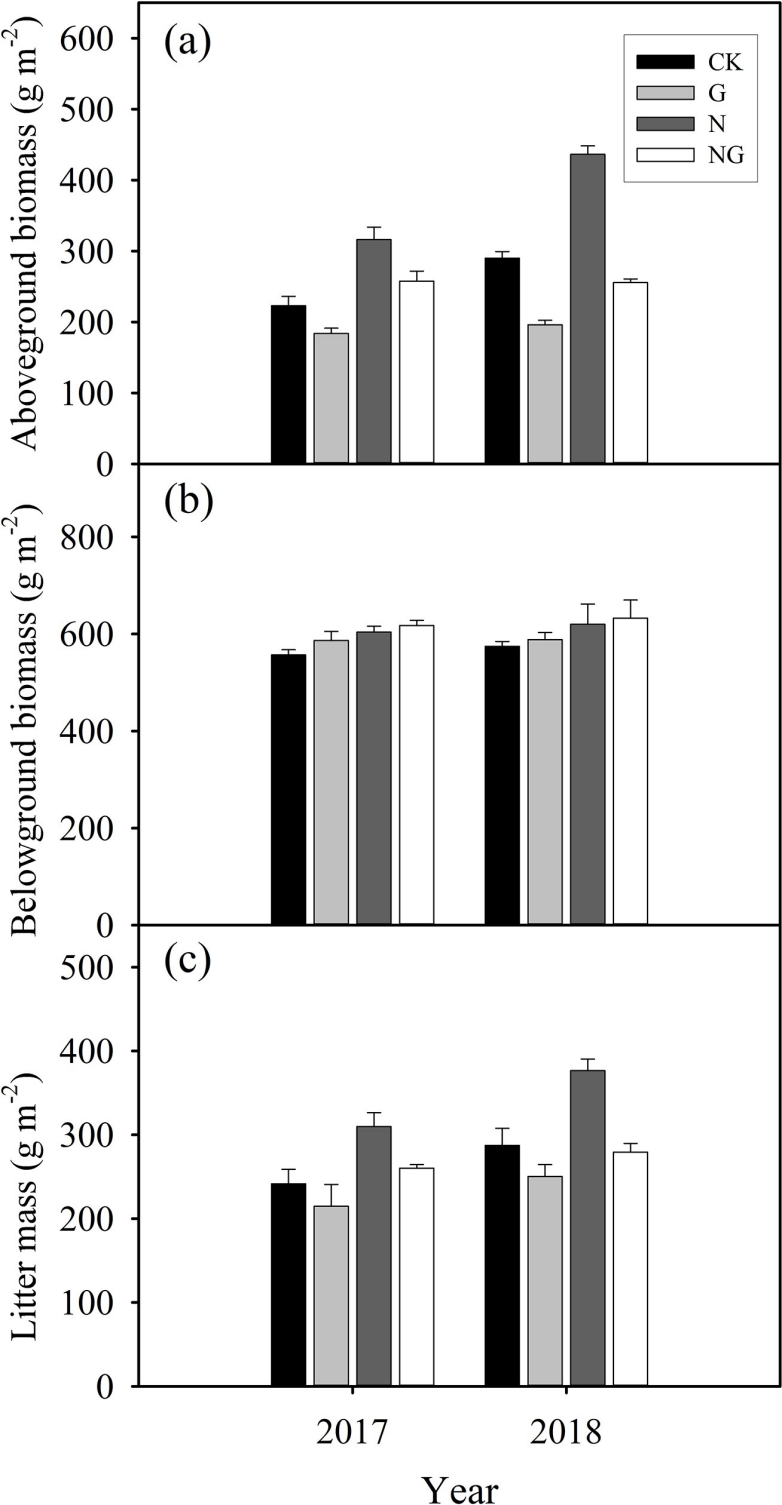
Effects of the experimental treatments (CK: control, G: grazing, N: nitrogen addition, NG: grazing and nitrogen addition) on aboveground biomass (a), belowground biomass (b) and litter mass (c) in 2017 and 2018. Data are reported as the arithmetic mean ± 1 standard error (n = 4).

Aboveground biomass (AGB) was stimulated by the N addition (Fig 3A; S1 Table); the AGB was enhanced by 41.9% and 50.3% in 2017 and 2018, respectively, relative to the control treatment. In contrast, grazing significantly (*P* < 0.01) reduced the AGB. The two studied growing seasons differed significantly (*P* < 0.01) in AGB, with it was higher in 2018 than in 2017. Only the N addition had a significant impact on belowground biomass (BGB) (Fig 3B; S1 Table). Grazing significantly (*P* < 0.01) reduced litter mass, while the N addition significantly (*P* < 0.01) increased litter mass (Fig 3C; S1 Table). The litter mass was higher in 2018 than in 2017.

### Effects of grazing and nitrogen addition on the soil physiochemical parameters

**Fig 4:**
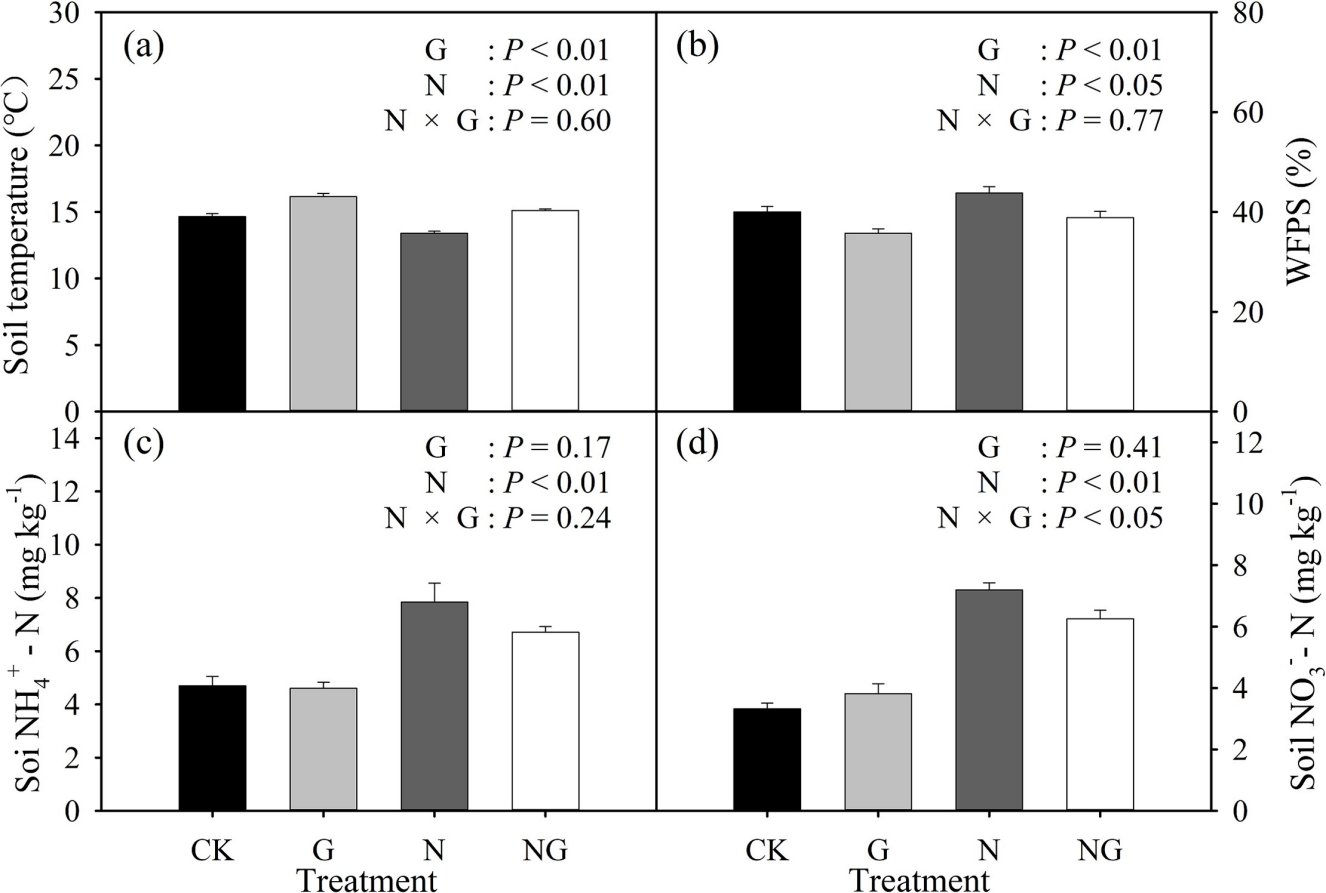
Effects of the experimental treatments (CK: control, G: grazing, N: nitrogen addition, NG: grazing and nitrogen addition) on 0–10 cm soil temperature (a), 0–10 cm soil water filled pore space (WFPS) (b), content of soil NH_4_^+^-N (c) and content of soil NO_3_^-^-N (d) from 2017 to 2018. Results of two-way ANOVA for the effects of grazing, N addition and their interactions on soil temperature, soil WFPS, soil NH_4_^+^-N and soil NO_3_^-^-N are provided. Data are reported as the arithmetic mean ± 1 standard error (n = 4).

Compared to the CK plots, the grazing treatment significantly (*P* < 0.01) increased the soil temperature by 1.5°C, whereas the N addition treatment significantly (*P* < 0.01) reduced the soil temperature by 1.25°C (Fig 4A). In contrast, the grazing treatment significantly (*P* < 0.01) reduced the soil WFPS by 10.6%, and N addition significantly (*P* < 0.01) increased it by 9.5% (Fig 4B) relative to the CK treatment. There were no significant interactive effects between grazing and N addition on the soil temperature and soil WFPS (Fig 4A & 4B).

Compared with the CK treatment, N addition significantly (*P* < 0.01) enhanced soil NH_4_^+^-N and soil NO_3_^-^-N contents, whereas the grazing treatment had no significant effects on soil NH_4_^+^-N and NO_3_^-^-N contents. There were significant (*P* < 0.05) interactive effects between grazing and N addition on soil NO_3_^-^-N content, but not on soil NH_4_^+^-N content (Fig 4C & 4D).

### Dependence of CH_4_ flux on plant biomass and soil parameters

**Fig 5:**
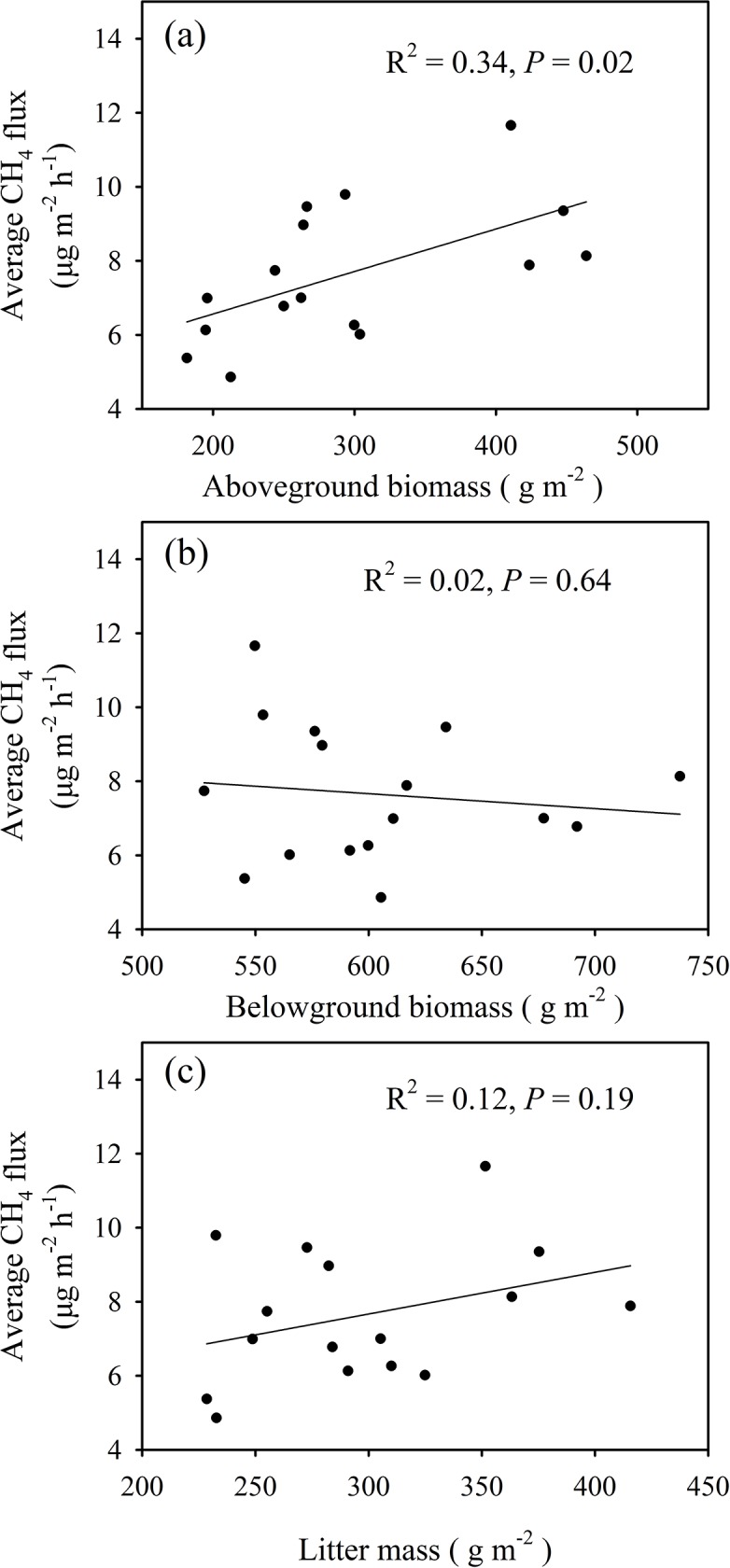
Dependence of average CH_4_ flux on (a) aboveground biomass (AGB), (b) belowground biomass (BGB) and (c) litter mass in 2018. Values of *R*^2^ and *P* are provided.

**Fig 6:**
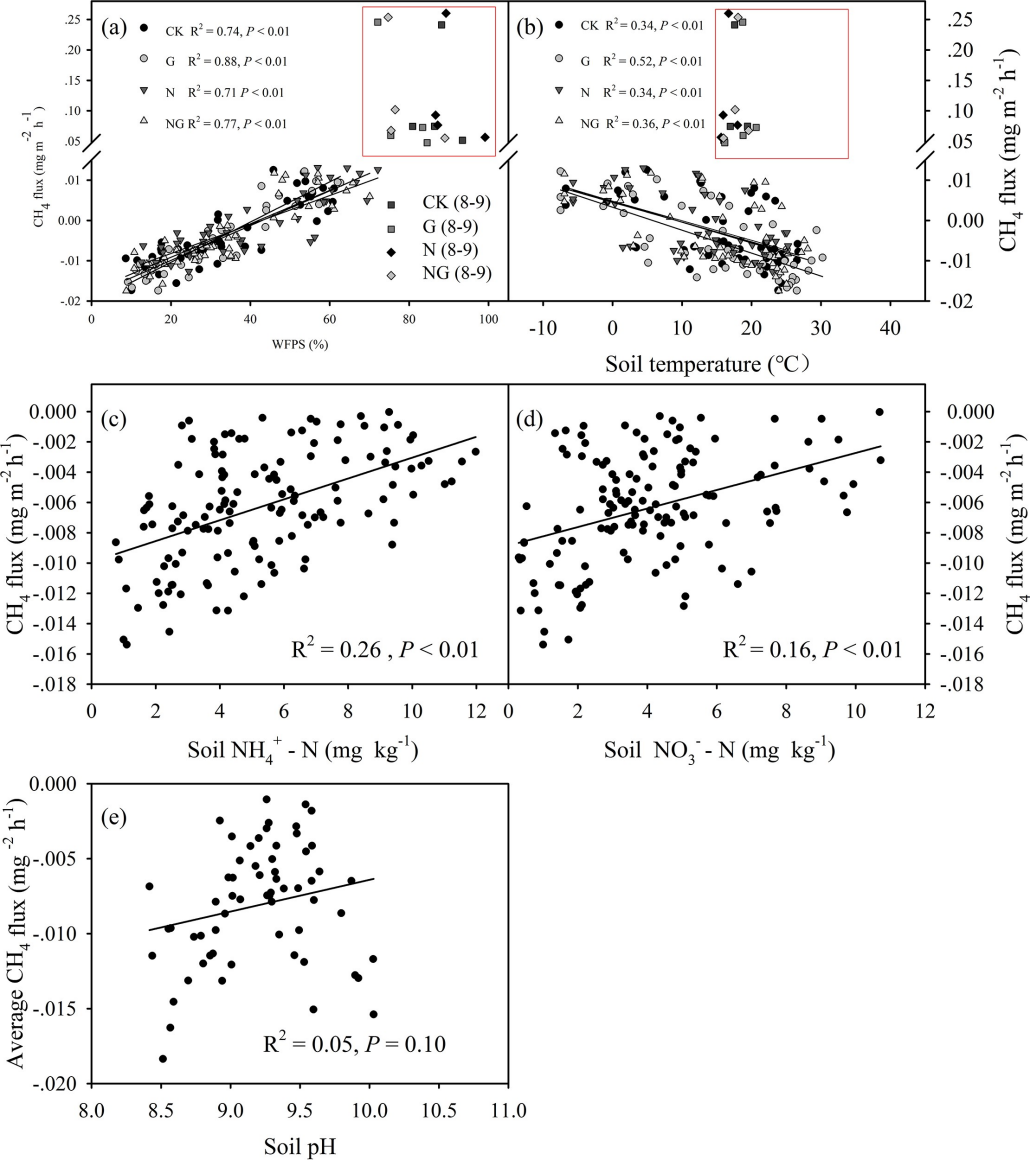
Dependence of CH_4_ flux on (a) soil water filled pore space (WFPS) and (b) soil temperature for each of the four experimental treatments (CK: control, G: grazing, N: nitrogen addition, NG: grazing and nitrogen addition). The CH_4_ fluxes (enclosed in a red square) from August to September in 2017 were not included in the regression analysis. Dependence of CH_4_ flux on (c) soil NH_4_^+^-N and (d) soil NO_3_^-^-N and (e) soil pH across the four treatments. Values of *R*^2^ and *P* are provided.

Average CH_4_ fluxes were positively correlated with aboveground biomass (*R*^2^ = 0.34, *P* = 0.02) (Fig 5A). However, the relationships between average CH_4_ flux and belowground biomass (*R*^2^ = 0.02, *P* = 0.64) and litter mass (*R*^2^ = 0.12, *P* = 0.19) in 2018 (Fig 5B & 5C) were not statistically significant. The soil CH_4_ fluxes were positively correlated with the soil WFPS (*R*^2^ = 0.71–0.88, *P* < 0.01) at 0–10 cm depth and negatively correlated with the soil temperature (*R*^2^ = 0.34–0.52, *P* < 0.01) at 0–10 cm depth from 2017 to 2018; the exceptions were the sampling dates that were strongly influenced by the extreme precipitation event (Fig 6A & 6B). CH_4_ fluxes were positively correlated with soil NH_4_^+^-N (*R*^2^ = 0.26, *P* < 0.01) and soil NO_3_^—^N (*R*^2^ = 0.16, *P* < 0.01) (Fig 6C & 6D). There was no significant (*P* = 0.10) relationship between soil pH at 0–10 cm depth and average CH_4_ flux (Fig 6E).

## Discussion

### Soil CH_4_ fluxes

Although CH_4_ uptake (negative flux) was occasionally found in all treatments, the average CH_4_ flux and cumulative CH_4_ flux were positive, indicating that our study site acted as a net source for atmospheric CH_4_ during the experimental period (Fig 2A–2D). This result contrasts with the results of most studies in grasslands in China [38]. The average CH_4_ fluxes in the four treatments ranged from 5.8 μg m^-2^ h^-1^ to 9.3 μg m^-2^ h^-1^ (Fig 2C), which were significantly higher than fluxes reported in typical grasslands in Inner Mongolia [39] and alpine steppe grasslands on the Tibetan Plateau [40]. This inconsistency may be attributed to the occurrence of an extreme precipitation event that occurred in August 2017. The extreme precipitation event strongly altered the soil WFPS and soil temperature, which promoted methanogenic activity and shifted the ecosystem from a CH_4_ sink to a source. In a previous study, Petrakis *et al*. (2017) reported that extreme inundation changed an ecosystem from a small CH_4_ sink to a small source of CH_4_ to the atmosphere [41], which highlights the importance of soil water content in controlling the balance of soil CH_4_ production and consumption [42]. Our observations showed that the potential of meadow steppe soils to act as CH_4_ sinks may decrease after extreme precipitation events.

### Effects of nitrogen addition on CH_4_ flux

Our results showed that N addition significantly increased soil CH_4_ emissions (Fig 2B & 2C), which is consistent with the results of most previous studies [11,43]. However, no effects of N addition on CH_4_ exchange were reported in subtropical plantation soils [44] or in degraded steppe soils [45]. N addition may influence CH_4_ flux by decreasing the uptake of CH_4_ or by increasing CH_4_ production. N addition enhanced the contents of soil NH_4_^+^-N and NO_3_^—^N, which are strong inhibitors of CH_4_ uptake [44]. First, NH_4_^+^ is a competitive inhibitor of CH_4_ oxidation due to the lack of specificity of methane monooxygenase (MMO) in methanotrophs [46]. Second, NH_4_^+^ oxidizes to the intermediate hydroxylamine (NH_2_OH) by CH_4_ monooxygenase or is further oxidized by other enzyme systems of methanotrophs to the end product of nitrite (NO_2_^-^). NO_2_^-^ is also produced via NO_3_^-^ reduction in anaerobic microsites. Hydroxylamine (NH_2_OH) and nitrite (NO_2_^-^) are toxic to methanotrophic bacteria [47]. Third, osmotic stress caused by the added nitrogen salt can suppress the activity of methanotrophs [48]. This inference was supported by the positive effects of N addition on the soil NH_4_^+^-N and NO_3_^—^N contents (Fig 4C & 4D) and the positive dependence of CH_4_ flux on the soil NH_4_^+^-N and NO_3_-N contents (Fig 6C & 6D). A previously published study suggested that CH_4_ uptake rate was sensitive to changes in pH and soil NO_3_^—^N, rather than NH_4_^+^-N [14]. However, we only detected a marginally significant relationship between soil pH and average CH_4_ flux (Fig 6E).

In addition to the effects on CH_4_ oxidation, N addition promoted biomass production and litter input (Fig 3C), which subsequently alleviated microbial C limitation [49]. As a result, the activities of methanogenic archaea were enhanced, and more CH_4_ was produced [49]. Moreover, we found average CH_4_ fluxes were positively correlated with aboveground biomass (Fig 5A). This likely occurred because increased aboveground biomass (i.e. greater canopy cover) reduces soil temperature via increased soil shading [50], which further decreases soil evaporation and increases soil moisture [51]. Grazing reduced the aboveground biomass while nitrogen addition increased aboveground biomass, so grazing increased soil temperature while nitrogen addition reduced soil temperature. This inference was supported by the positive dependence of soil CH_4_ flux on soil WFPS, and the negative correlation between soil CH_4_ flux and soil temperature.

### Effects of grazing on CH_4_ fluxes

Grazing is the most important human practice in grasslands and plays an important role in regulating the emission and uptake of greenhouse gases [17,18]. Grazing influences CH_4_ flux by altering aboveground and belowground productivity, litter input, microbial composition and soil physiochemical properties (soil temperature, soil moisture, soil nutrient content, etc.) but grazing effects depend highly on grazing intensity and duration. A previous study reported that grazing experiments with durations longer than 5 years had a significant effect on soil CH_4_ uptake, while experiments with durations less than 5 years had no effect on soil CH_4_ uptake [18]. In our study, the G and NG plots received moderate grazing by cattle in the growing season from 2016 to 2018, which was less than 5 years. However, grazing significantly decreased the average CH_4_ fluxes in our study (Fig 2B & 2C), which suggests that the CH_4_ fluxes in the studied meadow steppe are very sensitive to grazing disturbance.

Grazing is associated with shifts in plant biomass and soil structure and changes in soil temperature and soil moisture, all of which likely affect soil CH_4_ flux. First, grazing can alter soil temperature by increasing the radiant energy that reaches it, leading to higher soil temperature. Grazing reduced the surface vegetation coverage and litter biomass (Fig 3A & 3C) and enhanced soil temperature (Fig 4A), which likely had a greater impact on CH_4_ oxidation than on methanogen activities [32]. This inference was supported by the negative correlation between soil CH_4_ flux and soil temperature (Fig 6B). Methanotrophs are highly temperature sensitive [52], such that the rate at which they consume CH_4_ increases (i.e., a more negative flux rate) with increasing temperature [53]. Second, grazing decreased soil moisture (Fig 4B) by enhancing the soil temperature (Fig 4A) and evaporation [23]. Soil moisture influences CH_4_ flux by affecting microbial activities and influencing CH_4_ diffusion [40]. Grazing-induced reductions in soil moisture favour the diffusion of CH_4_ to methanotrophs in the subsurface soil, therefore increasing CH_4_ consumption. In line with previous work [54], we detected a strong positive dependence of soil CH_4_ fluxes on the soil WFPS at 0–10 cm depth (Fig 6A). Third, animal trampling disturbs the topsoil and decreases the diffusion of CH_4_ and oxygen from the atmosphere into the soil profile [55] or from the soil into the atmosphere, which can directly affect the soil CH_4_ flux. In the present study, the grazing treatment was carried out for only 3 years; therefore, trampling was unlikely to have significant impacts on soil compaction. Finally, decreases in aboveground biomass and litter mass production by grazing and the corresponding decline in C that is available as a substrate in the soil often result in lower soil CH_4_ emissions [56], which can lead to a decline in CH_4_ flux.

### Interactive effects of nitrogen addition and grazing on CH_4_ fluxes

Although N addition significantly increased the CH_4_ flux and grazing significantly decreased the CH_4_ flux, there were no significant interactive effects between N addition and grazing on CH_4_ flux during the experimental period (S1 Table). In general, N addition and grazing had opposite effects on the soil environmental conditions and vegetation parameters (Fig 4A & 4B). For example, soil temperature decreased in the N addition plots, whereas soil temperature increased in the grazing plots. Moreover, N addition increased AGB and litter, but grazing had the opposite effect. Therefore, when N addition and grazing were combined, no significant differences were detected between the NG and CK treatments for the aforementioned soil and vegetation parameters or for CH_4_ flux. Our results suggest that grazing management has the potential to offset the stimulating effects of N addition on CH_4_ emissions, which highlights the importance of land management for the estimation of global change factor effects on CH_4_ flux.

## Conclusions

Soil CH_4_ flux in the studied meadow steppe displayed strong seasonal variation and was highly influenced by the WFPS, topsoil temperature and inorganic N content. Due to the occurrence of an extreme rainfall event, the studied ecosystem was a net CH_4_ source during the experimental period. The N addition treatment significantly increased CH_4_ flux, whereas grazing significantly decreased CH_4_ flux. Grazing offset the stimulating effects of N addition on CH_4_ flux, and there was no difference in CH_4_ flux between the CK and NG treatments. Our results suggest that moderate grazing has the potential to reduce the negative impacts of N addition on CH_4_ flux and can increase the capacity of the soil CH_4_ sink in the studied meadow steppe. The present study highlights the importance of grassland management on the regulation of the response of ecosystem processes to global change stresses.

## Supporting information

S1 Table Results of three-way ANOVAs on the effects of year (Y), grazing (G) and nitrogen addition (N) on CH_4_ flux, soil water filled pore space (WFPS), soil temperature (ST), aboveground biomass (AGB), belowground biomass (BGB), litter mass from 2017 to 2018.
(DOC)
