# Supplementary material for: Grazing offsets the stimulating effects of nitrogen addition on soil CH4 emissions in a meadow steppe in Northeast China
Source: PLoS One. 2019 Dec 2;14(12):e0225862. doi: 10.1371/journal.pone.0225862 (PMC6886810; doi:10.1371/journal.pone.0225862)
Supplement: S1 Table — Results of three-way ANOVAs on the effects of year (Y), grazing (G) and nitrogen addition (N) on CH4 flux, soil water filled pore space (WFPS), soil temperature (ST), aboveground biomass (AGB), belowground biomass (BGB), litter mass from 2017 to 2018. (DOC) [file pone.0225862.s001.doc]

**Table 1． Results of three-way ANOVAs on the effects of year (Y), grazing (G) and nitrogen addition (N) on CH_4_ flux, soil water filled pore space (WFPS), soil temperature (ST), aboveground biomass (AGB), belowground biomass (BGB), litter mass from 2017 to 2018.**

| Factor | CH_4_ | | WFPS | | ST | | AGB | | BGB | | Litter | |
| --- | --- | --- | --- | --- | --- | --- | --- | --- | --- | --- | --- | --- |
|  | *F* | *P* | *F* | *P* | *F* | *P* | *F* | *P* | *F* | *P* | *F* | *P* |
| Y | 20.16 | **<0.01** | 0.08 | 0.78 | 66.91 | **<0.01** | 38.07 | **<0.01** | 0.64 | 0.43 | 9.87 | **<0.01** |
| G | 29.38 | **<0.01** | 12.97 | **<0.01** | 85.10 | **<0.01** | 135.33 | **<0.01** | 1.18 | 0.29 | 16.56 | **<0.01** |
| N | 13.09 | **<0.01** | 5.83 | **<0.05** | 45.93 | **<0.01** | 135.44 | **<0.01** | 6.79 | **<0.05** | 20.34 | **<0.01** |
| Y × G | 2.25 | 0.15 | 4.81 | **<0.05** | 4.67 | **<0.05** | 30.57 | **<0.01** | 0.07 | 0.80 | 2.69 | 0.11 |
| Y × N | 0.37 | 0.55 | 0.01 | 0.94 | 1.80 | 0.19 | 1.44 | 0.24 | 0.04 | 0.85 | 0.25 | 0.62 |
| G × N | 0.07 | 0.79 | 0.91 | 0.35 | 1.28 | 0.27 | 11.03 | **<0.01** | 0.07 | 0.79 | 4.68 | **<0.05** |
| Y × G × N | 0.01 | 0.91 | 0.02 | 0.88 | 0.79 | 0.38 | 4.40 | **<0.05** | 0.05 | 0.82 | 0.15 | 0.71 |
